# Projected shifts in loggerhead sea turtle thermal habitat in the Northwest Atlantic Ocean due to climate change

**DOI:** 10.1038/s41598-021-88290-9

**Published:** 2021-04-23

**Authors:** Samir H. Patel, Megan V. Winton, Joshua M. Hatch, Heather L. Haas, Vincent S. Saba, Gavin Fay, Ronald J. Smolowitz

**Affiliations:** 1 Coonamessett Farm Foundation, 277 Hatchville Road, East Falmouth, MA 02536 USA; 2 School for Marine Science and Technology, University of Massachusetts Dartmouth, 836 S Rodney French Blvd, New Bedford, MA 02744 USA; 3 Atlantic White Shark Conservancy, 235 Orleans Road, North Chatham, MA 02650 USA; 4 Northeast Fisheries Science Center, National Marine Fisheries Service, National Oceanic and Atmospheric Administration, 166 Water Street, Woods Hole, MA 02543 USA; 5 Geophysical Fluid Dynamics Laboratory, Northeast Fisheries Science Center, National Marine Fisheries Service, National Oceanic and Atmospheric Administration, Princeton University Forrestal Campus, 201 Forrestal Road, Princeton, NJ 08544 USA

**Keywords:** Animal migration, Marine biology, Climate-change ecology, Projection and prediction

## Abstract

It is well established that sea turtles are vulnerable to atmospheric and oceanographic shifts associated with climate change. However, few studies have formally projected how their seasonal marine habitat may shift in response to warming ocean temperatures. Here we used a high-resolution global climate model and a large satellite tagging dataset to project changes in the future distribution of suitable thermal habitat for loggerheads along the northeastern continental shelf of the United States. Between 2009 and 2018, we deployed 196 satellite tags on loggerheads within the Middle Atlantic Bight (MAB) of the Northwest Atlantic continental shelf region, a seasonal foraging area. Tag location data combined with depth and remotely sensed sea surface temperature (SST) were used to characterize the species’ current thermal range in the MAB. The best-fitting model indicated that the habitat envelope for tagged loggerheads consisted of SST ranging from 11.0° to 29.7 °C and depths between 0 and 105.0 m. The calculated core bathythermal range consisted of SSTs between 15.0° and 28.0 °C and depths between 8.0 and 92.0 m, with the highest probability of presence occurred in regions with SST between 17.7° and 25.3 °C and at depths between 26.1 and 74.2 m. This model was then forced by a high-resolution global climate model under a doubling of atmospheric CO_2_ to project loggerhead probability of presence over the next 80 years. Our results suggest that loggerhead thermal habitat and seasonal duration will likely increase in northern regions of the NW Atlantic shelf. This change in spatiotemporal range for sea turtles in a region of high anthropogenic use may prompt adjustments to the localized protected species conservation measures.

## Introduction

Warming ocean temperatures due to climate change are already having a measurable impact on ecological processes^1^. An emerging body of research has documented distribution shifts^2^, phenological changes to seasonal migration and reproduction^3^, and trophic mismatch^4^ in a wide variety of marine taxa. All of these changes increase the difficulty of managing commercially valuable marine species^5^ and protecting endangered and threatened animals^6^.

Understanding species distribution and habitat preferences are becoming fundamental components to developing effective resource management and conservation strategies^7,8^. Fisheries bycatch is one of the most serious threats to sea turtles around the world^9,10^. Attempts to mitigate bycatch levels are often based on an understanding of when and where a species occurs over time and how interactions occur with the fishing gear^11,12^. With the advent of time/area closures in fisheries management, more research is being conducted to understand the spatio-temporal nature of by-catch species^11^. In the Pacific, fisheries interactions with loggerhead sea turtles (*Caretta caretta*) have resulted in temporary area closures, and vessels must comply with stringent regulations to prevent the incidental capture of this species^13^. While these types of regulations have resulted in reduced bycatch of both loggerhead and leatherback sea turtles (*Dermochelys coriacea*)^14^, they will need to be continually modified to account for climate change.

Sea turtles, including the loggerhead, are susceptible to climate and ecosystem changes, particularly those associated with temperature. This has most commonly been documented with regards to sea turtle reproductive biology; previous studies have found that nesting phenology, hatchling sex ratios, and various metrics of nesting success can all be affected by even slight changes (< 3 °C) in ocean and air temperature [e.g.^15–17^]. In terms of marine distribution, habitable temperature ranges are broad, with loggerhead sea turtles observed throughout the NW Atlantic shelf region in waters with sea surface temperature (SST) ranging from 7°to 30 °C^18^. In a smaller study on loggerheads at the southern edge of the NW Atlantic shelf region, Coles and Musick^18^ found that the available thermal range (4.9°–32.2 °C) was broader than the occupied range (13.3°–28.0 °C), indicating that loggerheads at-sea likely stay within a preferred temperature envelope. Many marine species within the region are expected to shift their distribution to remain in preferred thermal habitat^19^. We hypothesize that loggerheads will do so similarly as the climate warms.

In the marine realm, species distribution modelling has been limited by the availability of species occurrence data and relevant environmental data^20^. Satellite telemetry has been used to monitor marine animals for over 35 years^21^. However, due to the cost prohibitive nature of these technologies, it is rare for a single population to be studied over many consecutive years^22–24^. As a result, relationships between sea turtle distribution and oceanographic variables have been based on relatively small, short term telemetry studies or opportunistic data sources, like fisheries bycatch [e.g.^13,14,25^]. The increasing abundance and availability of information collected by remote sensing tools such as satellite relayed data loggers and long-term high-resolution environmental data means that species distribution models (SDM) can now more easily be compared with oceanographic variables^26–28^.

Projections from global climate models are regularly used to estimate long-term shifts in the distribution of marine species^3^. However, only a few studies have attempted to project, over a long-term, the climate change induced shifts in available marine habitat for sea turtles. Using a thermal range previously established by Hawkes et al.^25^ for loggerheads in the NW Atlantic, Witt et al.^29^ projected the change in the availability of suitable thermal habitat in the Atlantic through 2089 using the global climate model HadGEM1 (Hadley Centre Global Environmental Model, version 1). Witt et al.^29^ calculated annualized northern and southern extents at which 90% of SST in the Atlantic Ocean will remain above 15 °C as a threshold for loggerhead distribution. In the Pacific, Hazen et al.^26^ used a generalized additive model to estimate the relationships between sea turtle distribution and several oceanographic variables. SST and chlorophyll-a values from Earth system model GFDL ESM 2.1 (Geophysical Fluid Dynamics Lab Earth System Model 2.1) were used to project the potential change in available ocean habitat through 2100^26^. This SDM provided a more direct correlation between the species’ distribution and the projected available habitat^30^.

**Figure 1:**
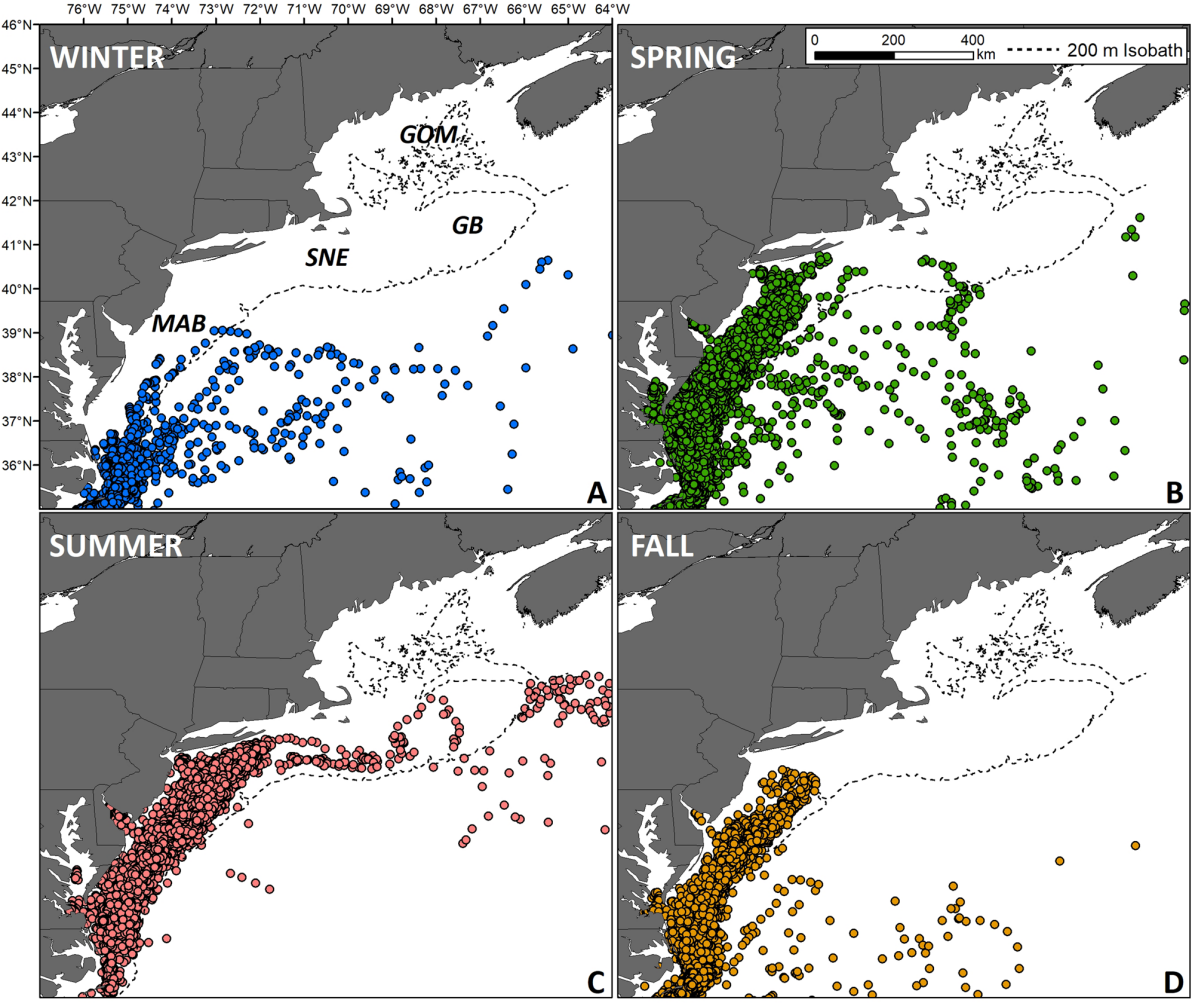
Reconstructed tracks from 196 loggerhead sea turtles satellite tagged between 2009 and 2018 within the northwest Atlantic. Dashed lines denote the 200 m bathymetric contour. GOM = Gulf of Maine, GB = Georges Bank, SNE = Southern New England, and MAB = Middle Atlantic Bight. Maps prepared with ArcMap 10.8, www.esri.com.

The Middle Atlantic Bight (MAB), Southern New England (SNE), Georges Bank (GB), and the Gulf of Maine (GOM) are adjacent continental shelf regions of the NW Atlantic Ocean (Fig. 1) that support a number of large commercial fisheries, a high amount of commercial and recreational vessel traffic, and the majority of the United States (US) federal wind energy lease areas^31^. Based on aerial surveys, the MAB is also a seasonal foraging ground for ~ 40,000– ~ 60,000 juvenile and adult loggerheads. The South Atlantic Bight (SAB) region of the US, between North Carolina and central Florida, is home to ~ 500,000– ~ 1,000,000 loggerheads during the summer months^32^. The population values for the MAB may be an underestimate as stable isotope analysis and satellite telemetry data indicate that potentially 30–50% of loggerheads that nest and reside along the US eastern seaboard seasonally forage within the MAB^28,32,33^.

In this study, we used satellite tagging data collected from 2009 to 2018 to identify relationships between loggerhead occurrence, SST and depth and characterized their current bathythermal habitat. We projected potential climate-change related shifts in the distribution of loggerhead bathythermal habitat over the next 80-years using projections of SST for the region from a high-resolution global climate model. Overall, we suggest that the loggerhead marine bathythermal habitat will likely expand to the northern regions and increase in seasonal duration to earlier in the spring and later into the fall.

## Methods

### Loggerhead satellite tagging

All loggerheads were tagged between 2009 and 2018. The majority of turtles (n = 190) were tagged within the MAB between May and September. Two turtles were tagged in eastern GB and one was tagged off-shelf near southern MAB at the edge of the Gulf Stream, a fast moving current that flows along the US eastern seaboard pulling warm water from the Gulf of Mexico northward and eastward^34^. Three additional turtles were tagged off of the North Carolina coast in February. Curved carapace length, from anterior notch to posterior tip, was measured for each captured turtle. Most (n = 186) turtles were equipped with a satellite relay data logger manufactured by the Sea Mammal Research Unit at the University of St Andrews; and ten turtles were equipped with a Wildlife Computers SPOT tag. See Patel et al.^35,36^, Winton et al.^29^ and Crowe et al.^37^ for full details on capture and handling protocols, satellite tag parameterizations, and more details from the satellite tag data.

All fieldwork was approved by the US National Marine Fisheries Service (NMFS) Atlantic Institutional Animal Care and Use Committee and the US Endangered Species Act (ESA) Section 10(a)(1)(a). Work was conducted under ESA permits #14249 and #18526 issued to Coonamessett Farm Foundation, Inc., ESA permits #1576 and #16556 issued to the Northeast Fisheries Science Center and ESA permit #1551 issued to the Southeast Fisheries Science Center. All methods were carried out in accordance with approved guidelines and regulations.

### Data processing

Telemetered location data were processed following standard guidelines for sea turtle tracking data. Tracks of individual turtles were filtered using a Continuous Time Correlated Random Walk movement model fitted to location data using the software ‘Template Model Builder’^38^ in R^39^. Daily position estimates were interpolated from each movement model’s output^29,40,41^ to correct for the different transmission rates of each tag and to reduce autocorrelation in position estimates. Prior to fitting the movement model, all location coordinates were re-projected into the oblique Mercator center projection centered on 35.0°N, 75.0°W using R package ‘rgdal’^42^ and a speed filter with a max speed of 5 km/hr was applied to remove errant telemetered locations^43^.

For analysis, loggerhead locations were aggregated over the NMFS Atlantic Marine Assessment Program for Protected Species (AMAPPS) spatial grid that has a 10-km resolution^29^. For model fitting, we used loggerhead location estimates from continental shelf waters (depths < 200 m) between 33.5°N and 41.6°N, which encompasses the MAB as delineated by NMFS statistical areas^44^ and corresponds to the highest density area traversed by satellite-tagged loggerheads^29^. Although on occasion loggerheads were tracked in waters deeper than 200 m and north of 41.6°N latitude, those locations were removed from this study due to the low sample size and the higher incidences of fisheries interactions occurring on shelf waters^45^ Locations were binned by month to match the resolution of climate projections^46^ and aggregated over the 10-km resolution AMAPPS spatial grid. The AMAPPS grid was bounded by the coastline to constrain loggerhead space use to the ocean.

### Characterizing the bathythermal range of loggerheads

Although we understand loggerheads are likely influenced by a large range of environmental variables, our goal was to investigate how the distribution of loggerheads may change in response to warming water temperatures associated with climate change. To model spatial variation in the occurrence of tagged loggerheads and identify the proportion of the observed variation related to water temperature, generalized linear models (GLMs) were applied to estimate the relationship between the probability of loggerhead presence, SST, and depth. We modeled the occurrence, *y*_*it*,_ (0 = absent, 1 = present), of tagged turtles in grid cell *i* during time step *t* as the outcome of a Bernoulli random variable:

$${y}_{it}\sim {\text{Bernoulli}}\left({p}_{it}\right),$$

where *p*_*it*_ is the probability that a tagged turtle was present*.* We modeled *p*_*it*_ as a function of SST and depth as:

$$\mathrm{logit}\left({p}_{it}\right)={\beta }_{0,}+{\beta }_{1}{\mathrm{SST}}_{it}+{\beta }_{2}{SST}_{it}^{2}+{\beta }_{3}{Depth}_{it}+{\beta }_{4}{Depth}_{it}^{2},$$

where the logit link function constrains *p*_*it*_ from 0 to 1, *β*_*0*_ is an intercept term; *β*_1_ and *β*_2_ represent a quadratic effect of SST (which allows for a non-linear relationship); *β*_3_ and *β*_4_ a quadratic effect of bottom depth. Depth data for daily loggerhead locations were obtained from the gridded ETOPO1 bathymetry data set^47^. For observed ocean temperature data (2009–2018), we used the Optimum Interpolation Sea Surface Temperature (OISST) product for the same time period of the turtle tracking. OISST is a combination of observations from different platforms (satellites, ships, buoys) and is produced at a 1/4° spatial resolution^48^. Daily OISST satellite composites were obtained from the NOAA CoastWatch Program (http://coastwatch.pfeg.noaa.gov/erddap/griddap/) using functions available in the R package ‘rerrdap’^49^ and averaged together to create monthly climatologies to match the output of the climate model projections. These composites were then up-sampled to align with the AMAPPS grid by simple averaging.

All model variants were fit via maximum likelihood methods using the package ‘Template Model Builder’^38^. All parameters were treated as fixed effects; the final gradient value for parameters and the hessian matrix were inspected for each model fit to confirm convergence. We used the Akaike information criterion (AIC)^50^ and the percent deviance explained^51^ for model selection. Given the small number of explanatory variables considered, we used a forward, step-wise selection approach to identify the most parsimonious combination of regression terms^52^. Individual terms were retained in the model if their inclusion resulted in a lower AIC and increased the proportion of the deviance explained relative to the best-fitting model from the previous step. To assess the fit of the selected model and identify potential model misspecification, we examined standard residual diagnostic plots using normalized, randomized residuals^53^. Visualizations of model results were produced using functions available in the R package ‘tidyverse’^54^.

### Forecasting the distribution of loggerheads under climate change

To investigate how the distribution of loggerheads may shift under climate change, the selected model was fitted to long-term (80 year) projections of SST in the MAB, SNE, GB, and GOM, the most northern portion of loggerhead range within the western Atlantic Ocean^29^. Projections were based on a climate change scenario from the National Oceanic and Atmospheric Association’s (NOAA’s) high-resolution global climate model (CM2.6) as described and validated by Saba et al.^46^ for the Northwest Atlantic. Unlike most global climate models that have a warm bias due to misrepresentation of the position of the Gulf Stream, CM2.6 resolves the Gulf Stream, regional ocean circulation, and bathymetry of the Northwest Atlantic shelf^46^ much more realistically. Overall, CM2.6 outperformed all coarser models that were assessed^46^. Many studies that have projected marine species habitat shifts in the Northwest Atlantic have relied on this climate model^20,55–57^.

The SST output from CM2.6 represents a monthly deviation from a historical average derived from control simulations (deltas). The CM2.6 output was rasterized onto a 0.1° × 0.1° mesh and then synced to the AMAPPS grid. The SST deltas were then added to the mean monthly SST values for the observed time period. Along with depth, which we assumed remained constant, the projected monthly SST was used to project the probability of loggerhead presence from the MAB north to GOM within the continental shelf for 80 years conditioning on the fitted model. For visualization purposes, observed and projected data were grouped into seasons based on both general climate trends for the region and turtle habitat usage patterns^29,36,58^. The projected probability of presence was then averaged across years (10 and 20 year bins). January through March were grouped into winter, April through June to spring, July through September to summer; and October through December to fall.

### Quantifying climate-related shifts in distribution

To better understand and visualize the predicted changes in loggerhead occupancy (presence/absence) under climate change, we developed a binary classifier using the Index of Union (IU) approach to determine whether a cell would be occupied by a loggerhead turtle given the identified relationships^59^. This analysis was done using the R package “ROCR”^60^. In short, the IU approach attempts to find an optimal cut point (*c*) that correctly classifies the fitted continuous probabilities of loggerhead presence as a 1 (present) or 0 (absent). The optimal value of *c* is that which minimizes the IU criterion:

$$IU\left(c\right)= \left(\left|Se \left(c\right)-AUC\right|+ \left|Sp \left(c\right)-AUC\right|\right),$$

where *Se* is the sensitivity (true positives / (true positives + false negatives)), *Sp* is the specificity (true negatives / (true negatives + false positives)), and *AUC* is the Area Under the Receiver Operating Curve. The optimal cut-point was found to be *c* = 0.08 (i.e., predicted probabilities ≥ 0.08 were classified as 1, otherwise 0), with *Se* (*c* = 0.08) = 0.84, *Sp* (*c* = 0.08) = 0.70, and the *AUC* = 0.85. Using the optimal cut-point, we classified seasonal averages of presence probabilities by projected decade to identify cells that could be occupied by loggerhead turtles based on the bathythermal habitat associated with observed loggerhead occupancy patterns. We labeled the IU region classified to have loggerhead presence as the ‘core habitat’. The fraction of cells in the study area that could be occupied by loggerheads was then calculated to explore trends in projected occupancy over time. We also calculated the region with the highest probability of presence by taking the top 25% of the predicted habitat values^27^.

## Results

In total, 196 loggerheads from 2009 to 2018 were fitted with satellite tags (Table 1). Turtles were either late-stage juveniles or adults with a mean (± SD) curved carapace length of 80.0 ± 9.7 cm. We found no qualitative difference in the seasonal movement patterns between presumed late-stage juveniles and adults and as such pooled across life-stage for this analysis. Filtering the location estimates from these tags yielded 45,840 daily locations within the NW Atlantic (north of 33.5°N and west of 64°W, the approximate southern and western boundaries of the US northeast continental shelf Large Marine Ecosystem^61^), of which 44,865 daily locations occurred on the continental shelf in the MAB and were used for model fitting.

**Table 1:** Summary information for satellite tag deployments (CCL = curved carapace length from notch to tip).

Year	Tags deployed	Mean CCL	SD CCL
2009	2	71.8	7.4
2010	14	77.8	9.2
2011	26	79.1	7.8
2012	30	81.9	8.7
2013	16	79.2	13.4
2014	18	78.2	9.8
2015	10	78.7	12.4
2016	21	80.4	8.6
2017	24	78.5	12.0
2018	35	82.7	8.4
Mean	19.6	80.0	9.7

**Figure 2:**
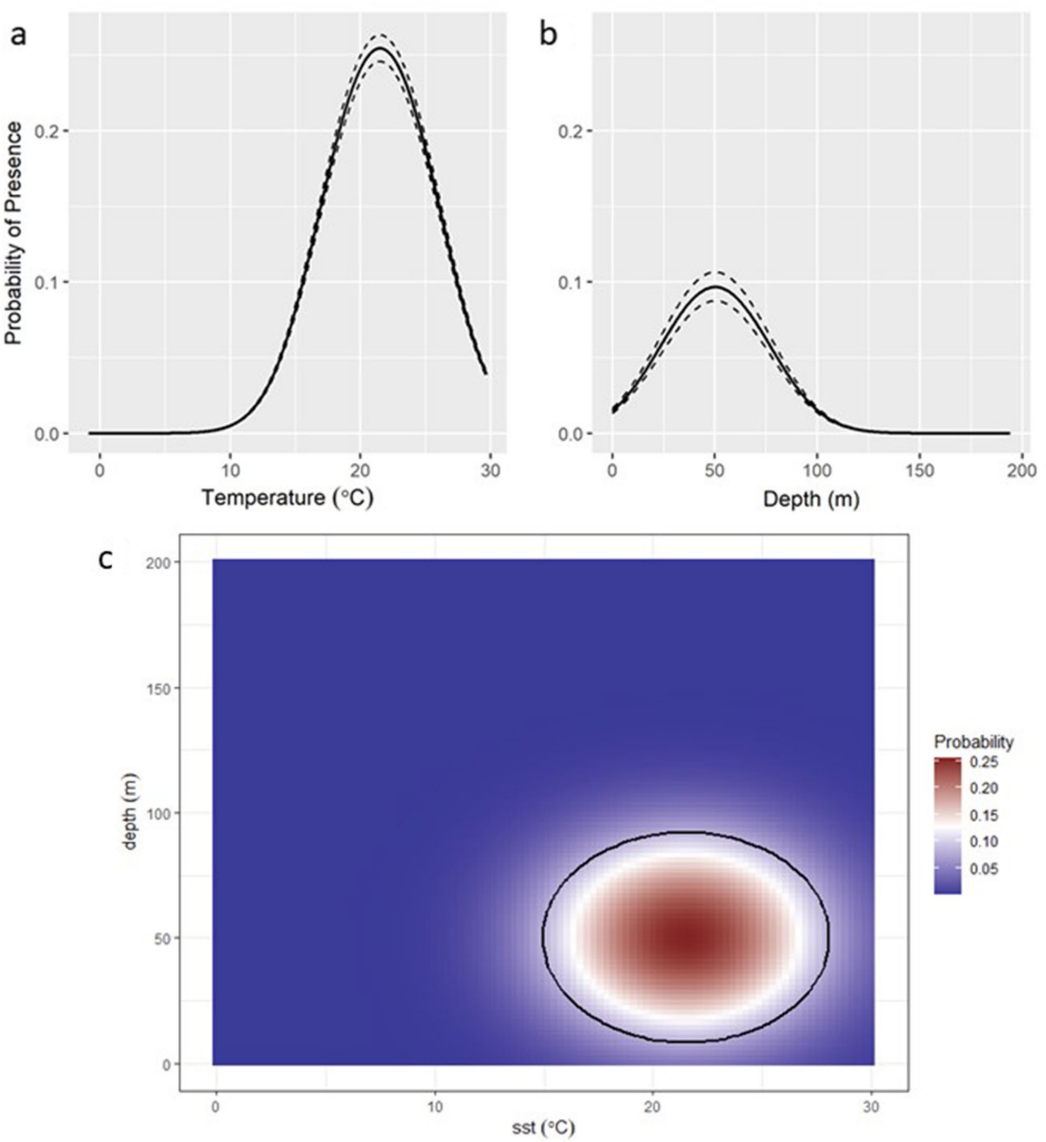
Probability of presence of loggerheads in relation to (**a**) sea surface temperature (SST) and (**b**) bottom depth. Dashed lines indicate 95% confidence intervals. The resulting core habitat as identified using the ‘Index of union’ is illustrated in (**c**), where the graph identifying the probability of loggerhead presence from observed data associated with the combined SST and depth ranges and the calculated core habitat (black circle).

Model selection for explanatory variables supported a relationship between loggerhead presence, SST, and depth. SST alone explained 15.4% of the variability in loggerhead presence, while including only bottom depth explained 4.1%. SST and depth combined explained 20.1% of the variability in loggerhead presence. Based on the fitted model, loggerheads occur at SST between 11.0° and 29.7 °C and at depths between 0 and 105.0 m (Fig. 2a,b). The overall predicted distribution for each month was consistent with the reconstructed tracks and indicated that the probability of loggerhead presence in the NW Atlantic shelf waters is highest from May through October. Portions of SNE and GB were estimated to have a higher probability of presence than the MAB during summer months.

Using the binary classifier resulted in a core habitat that consisted of temperatures between 15.0° and 28.0 °C and depths between 8.0 and 92.0 m (Fig. 2c). The highest probability of presence occurred in regions with SST between 17.7° and 25.3 °C, and depths between 26.1 and 74.2 m. More specifically, turtles tended to occupy regions of the MAB with SST closest to 21.5 °C at depths closest to 50 m.

**Figure 3:**
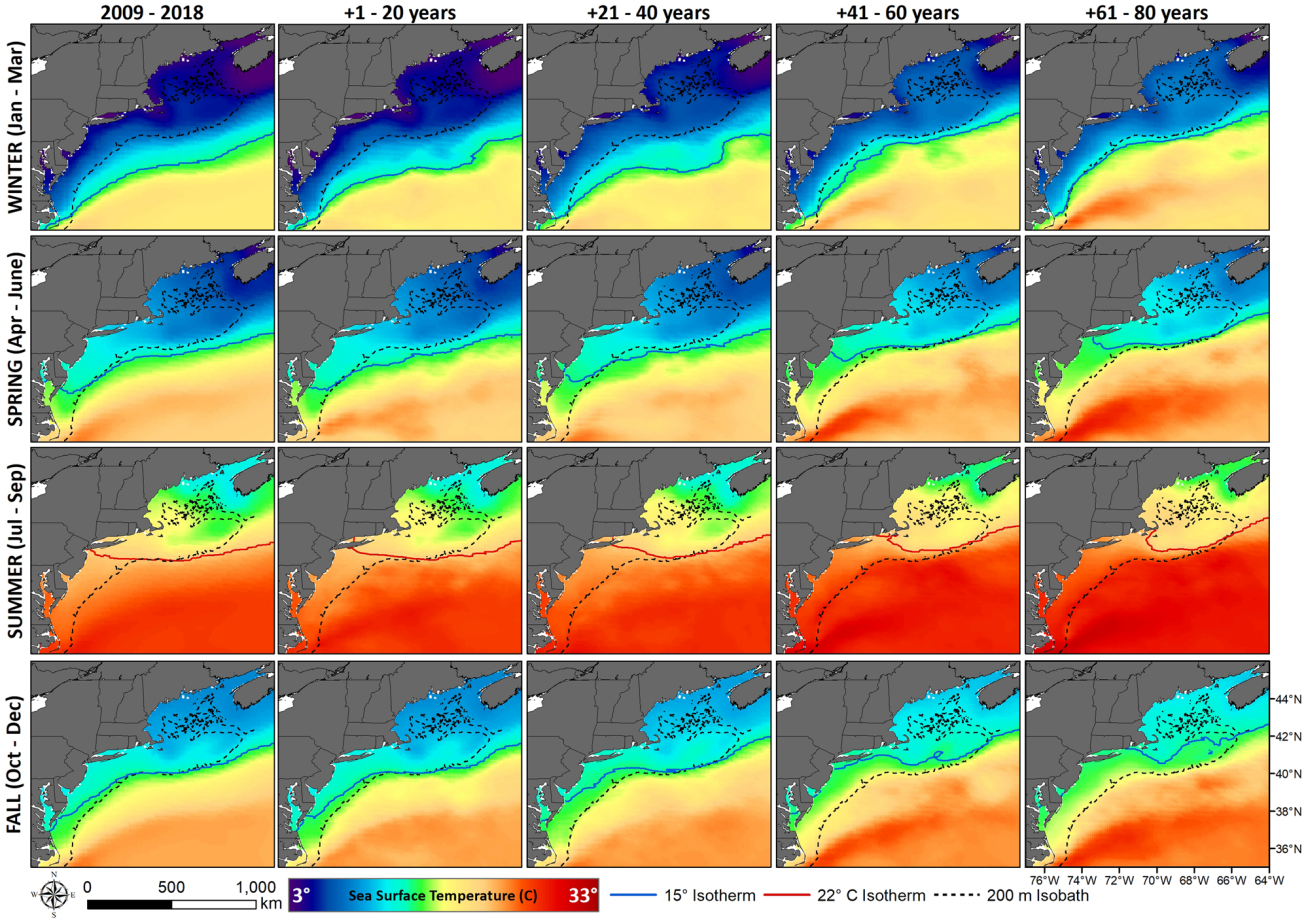
Seasonal maps of historical and projected sea surface temperature in the northwest Atlantic. The north- and shore-ward movement of the Gulf Stream is expected to increase warming within shelf waters. Maps prepared with ArcMap 10.8, www.esri.com.

**Figure 4:**
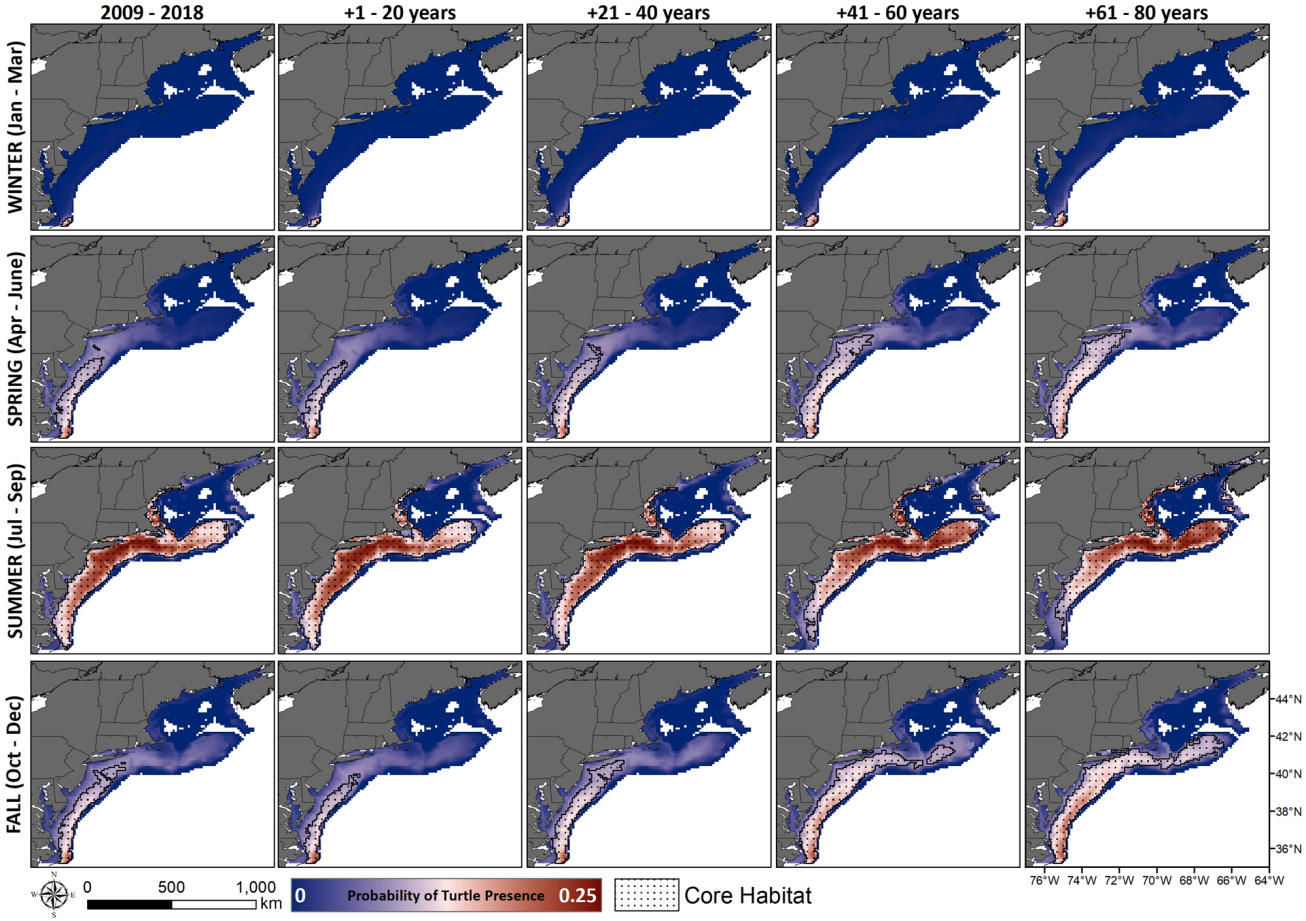
Seasonal maps of probability of turtle presence and core habitat based on observed and projected sea surface temperatures (SST) using the CM2.6 model. Color ramp matches Fig. 2c and indicates the probability of presence based on SST and depth. Maps prepared with ArcMap 10.8, www.esri.com.

**Figure 5:**
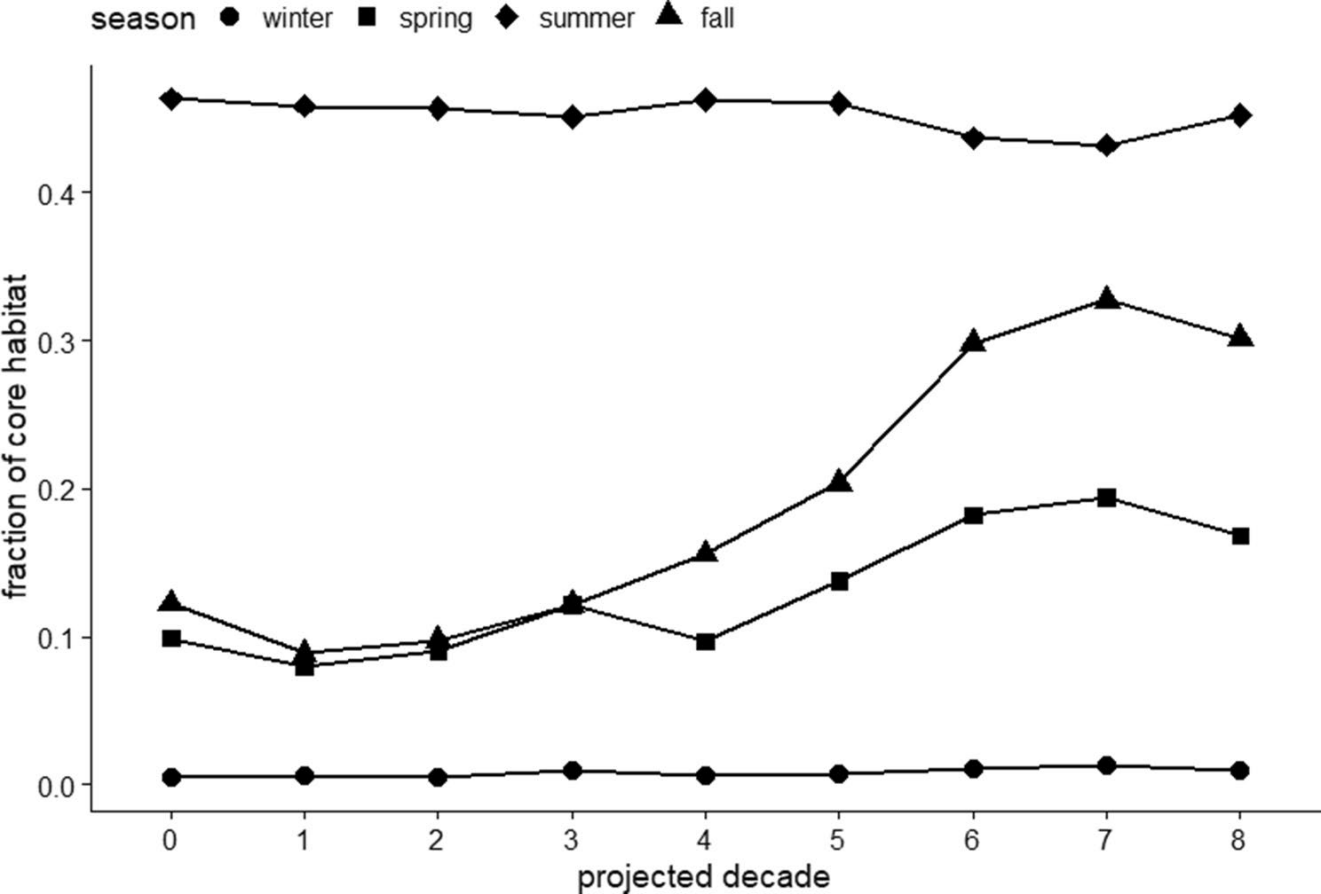
Change in fraction of the NW Atlantic shelf region identified as core habitat for loggerheads across the projected 80 years binned by decade. Spring and fall are projected to have the largest change. Decade ‘0’ refers to the observed data.

The CM2.6 model projected that warmer SST would push farther inshore and north through all seasons (Fig. 3). Mean SST within the shelf region is expected to gradually increase in the first 40 years, then intensify over the following 40 years. The probability of presence for loggerheads in the MAB is projected to increase from the observed May–October season, to an April–December season within 20–60 years, encompassing the entire spring, summer and fall seasons (Fig. 4). In particular, fall is expected to have the largest increase in available thermal habitat for loggerheads, followed by spring (Fig. 5). Minimal changes in winter are expected in terms of available suitable habitat over the next 80 years, while available habitat in the summer is expected to slightly decline as the most southern portions of the MAB warm beyond the range of our established temperature envelop.

## Discussion

Based on the current relationship between loggerhead occurrence and SST, projected increases in SST as a result of climate change will likely result in an expansion of potentially suitable loggerhead habitat in the NW Atlantic. While the CM2.6 model projects a ~ 3 °C increase in mean SST for all seasons within 60–80 years, this increase in SST is not expected to be uniform—the GOM is expected to warm faster than the more southern regions^46^. Shelf waters in the MAB are typically cooler than offshore waters with these two water masses bounded by the Slope Sea, a narrow band of ocean along the continental slope between the shelf and the Gulf Stream^62^. However, CM2.6 projects a change to these conditions with the southward flowing cool Arctic waters of the Labrador Current retreating along with a north- and shore-ward shift of the Gulf Stream, allowing warmer water to enter the NW Atlantic shelf region^46^. These oceanographic features are expected to provide continued suitable bathythermal habitat for loggerheads in the MAB, SNE and GB, and may also expand the range of suitable habitat beyond the observed season^29,58^ and northward into GOM.

Our results matched well with previous research attempting to establish the bathythermal range for loggerheads in the region. Hawkes et al.^26^ found that satellite tagged loggerheads occupied a similar range of temperatures (12.8°–30.4 °C) and depths (0.25–104.4 m), while Hawkes et al.^63^ updated these results with additional telemetry data and narrowed the ranges to SST = 18.2°–29.2 °C and depth = 3–89 m. Additionally, Mansfield et al.^64^ identified that tracked loggerheads that stayed within the neritic zone of the NW Atlantic experienced an SST range between 9.0° and 29.3 °C across all seasons. During the summer the MAB experiences a sharp thermocline with the formation of a Cold Pool water mass that is ~ 15 °C cooler than surface waters and occurring near-bottom at depths between 30 and 70 m^65^. Loggerheads regularly forage within the Cold Pool^36^ and those that remain in the shelf waters of the southern US regularly inhabit environments with a much higher SST than observed in the MAB^66^. In addition, with our tagged turtles inhabiting the northern edge of western Atlantic loggerhead distribution^29^, the range of SSTs and depths reported here may be slightly narrower than the temperature and depth ranges in which these animals are able to thrive. Loggerhead response may thus not match the rate of the projected northward shift in their available thermal envelope or their movements may be driven be other correlating factors^27^.

Despite our use of only two explanatory variables (SST and depth), our model results showed similar patterns of loggerhead distribution to sightings, strandings, and bycatch data, with slight variations due to the unique practices of each fishery. Braun-McNeill et al.^67^ found that 11 °C was a conservative minimum SST threshold that aligned well with sea turtle distribution in the NW Atlantic shelf region from ten years of sightings, strandings and bycatch data. Swimmer et al.^14^ identified that loggerheads were most often caught in long lines when SST ranged between 18° and 24 °C and hook depth was 22 m or shallower; however, these results included a much larger portion of the greater Atlantic Ocean. Gillnet bycatch between Massachusetts and North Carolina, having occurred nearly year-round, was within a broader range of SST and depth (SST = 8.6°–27.8 °C; depth = 1.8–76.8 m^68^). Observed bycatch in scallop dredges was limited to SST between 18° and 25 °C and depths of 36–68 m^69^. These values from the scallop fishery aligned closest to our ranges for highest probability of turtle presence (SST = 17.7°–25.3 °C and depths = 26.1–74.2 m) because by-caught turtles in the scallop fishery had a high spatiotemporal overlap with when and where we caught the majority of our tracked loggerheads. Simultaneous integration of multiple data streams during statistical model estimation could help with more robust characterization of habitat for marine species in addition to this corroborative evidence, particularly for cases with incomplete and imperfect data resolution and could be a target for future research.

We built upon projections calculated by Witt et al.^70^ of suitable loggerhead habitat by developing probability models with monthly projections. Witt et al.^70^ created annual projections using a 15 °C threshold and suggested that for 90% of the year the MAB and areas north are unsuitable habitats for loggerheads, even as ocean temperatures warm. However, Witt et al.^70^ added that during summer months, loggerheads would regularly forage farther north than their annualized habitat suitability contours. Results of our winter projections matched well with annualized contours from Witt et al.^70^, indicating that loggerheads would have a very low probability of entering the MAB during this season, remaining farther south, or potentially in warmer offshore waters. However, throughout the remainder of the year, we projected that the loggerhead thermal habitat envelope would expand into MAB shelf waters earlier in the spring, continue moving north beyond the observed range in SNE and GB, and retreat south later in the fall. This corresponds closely with the trend of the spring and fall 15 °C SST threshold, with this isotherm continuing north in both shelf and offshore waters throughout the next 80 years.

Although observed data from aerial surveys indicates that SNE and GB are already suitable for loggerheads during portions of the year^18^, turtles are likely not as abundant farther north due to the shorter thermal window and the existing availability of prey resources in the MAB. With the projected increased thermal window, loggerheads may have more time to explore and actively forage within the northern shelf regions, while reducing competition for prey resources in the more heavily populated MAB, creating higher value to the longer distance migration^71^.

In general, there are likely other shifts in loggerhead distribution that could occur as environmental conditions in the NW Atlantic change. For example, rising SST is expected to contribute to an increase in hurricane activity and intensity within this region^72^. Crowe et al.^37^ identified that some loggerheads in the MAB initiated their southern migrations well in advance of the established seasonal trends due to the passing of Hurricane Irene. As a result, the seasonal movement patterns we have projected may be disrupted by an increase in hurricane activity. In addition, for this study we focused on foraging within the continental shelf; however, if conditions become unsuitable for loggerheads in the MAB, turtles may forage off-shelf for extended periods of time instead of migrating north^73,74^. Our tagging data do indicate that loggerheads venture off-shelf on rare occasions, and adult and sub-adult loggerheads have been tracked foraging in pelagic environments within the NW Atlantic and throughout the world^64,74–78^. In the MAB, loggerheads have been observed foraging pelagically on jellyfish^79,80^, and the off-shelf regions adjacent to GOM, GB, MAB and SNE in the Northwest Atlantic are known migratory corridors and feeding grounds for leatherback turtles, obligate jellyfish foragers^81,82^.

As the thermal habitat in the MAB through GB shelf region changes, this will also likely cause shifts in prey densities and species compositions. Using the same CM2.6 global climate model, Kleisner et al.^20^ described the shifts in available thermal habitats for over 30 commercially valuable marine species within the NW Atlantic continental shelf. In general, Kleisner et al.^20^ projected the expansion of available thermal habitats for southern species, and the reduction in available thermal habitats for northern species during the spring and fall seasons. Amongst these species, the vulnerability of the Atlantic sea scallop (*Placopecten magellanicus*) to climate change may provide an indication of how turtles may shift habitat usage. Atlantic sea scallops are a known prey item for loggerheads^83^ and there is generally a high overlap between loggerhead and sea scallop distribution in the MAB based on preferred depth range^84^. Recent research by Rheuban et al.^85^ has found that each scallop stock (MAB and GB) may react differently to climate change and that the more northern GB population may be slightly buffered from negative impacts due to the different mechanisms for larval recruitment between the stocks. However, using changes in ocean temperature and salinity from CM2.6, Tanaka et al.^55^ projected substantial scallop habitat declines throughout the MAB and GB but increased habitat in coastal GOM. As a result, the benthic community could substantially change in the MAB, potentially causing loggerheads to seek other, perhaps more northerly, shelf habitats for prey resources.

Climate based shifts in the distributions of sea turtles and commercially harvested species may change future patterns of bycatch. Changes to loggerhead range and seasonality may create spatial overlap with fisheries that have not previously needed sea turtle conservation measures. In the NW Atlantic, Kleisner et al.^20^ identified that most commercial fisheries would likely have to change their practices due to climate change, as distances and directions from ports to fishing grounds are expected to substantially change due to projected shifts in commercial species’ distributions. For example, the Atlantic sea scallop fishery developed gear modifications, (Turtle Deflector Dredge and Turtle Chains^83^) to reduce the bycatch of sea turtles and mandated these measures for boats fishing in the MAB, specifically west of − 71°W, from May through November^86^. Our model projects that within the next 20–40 years, loggerheads could forage within the NW Atlantic shelf region outside the spatial and temporal range these scallop gear modifications are required. Because the scallop industry has already developed a dredge effective at reducing turtle bycatch, adjusting the gear to remain efficient in the more northern scalloping grounds and expanding its usage could be an effective solution with minimal economic impacts to the fishery^83^. However, northern fisheries that use pelagic long lines, trawls and gillnets have the potential to see increases in turtle bycatch if fisheries management does not adapt to projected environmental changes. For example, the bottom trawl fishery operating in the MAB, SNE and GB, from 2014 to 2018, had the highest number of estimated sea turtle interactions occur north of 39°N, which is farther north than in previous years^87^.

Overall, sea turtle seasonal habitat usage and distribution is certainly linked to a broader range of environmental variables beyond SST and depth^27^, as well as biological factors like the availability of prey resources^88^, reproductive cycles and life stage^89^. However, given the availability of data and what is known about loggerhead ecology in general, the type of SDM we present provides a reasonable assessment of the potential drivers for the distribution of this cohort of loggerheads^30^. To truly determine how climate change will impact these turtles will require continued monitoring, particularly in the MAB, SNE and GB. Our results can guide expectations for likely future turtle distributions and inform discussions to plan for climate change-resilient conservation measures.
